# Recent updates and developments to plant genome size databases

**DOI:** 10.1093/nar/gkt1195

**Published:** 2013-11-27

**Authors:** Sònia Garcia, Ilia J. Leitch, Alba Anadon-Rosell, Miguel Á. Canela, Francisco Gálvez, Teresa Garnatje, Airy Gras, Oriane Hidalgo, Emmeline Johnston, Gemma Mas de Xaxars, Jaume Pellicer, Sonja Siljak-Yakovlev, Joan Vallès, Daniel Vitales, Michael D. Bennett

**Affiliations:** ^1^Laboratori de Botànica-Unitat Associada CSIC, Facultat de Farmàcia, Universitat de Barcelona, 08028 Barcelona, Catalonia, Spain, ^2^Jodrell Laboratory, Royal Botanic Gardens, Kew, Richmond, Surrey, TW9 3AB, UK, ^3^Departament de Biologia Vegetal, Facultat de Biologia, Universitat de Barcelona, 08028 Barcelona, Catalonia, Spain, ^4^Department of Managerial Decision Sciences, IESE Business School, Universidad de Navarra, 08032 Barcelona, Catalonia, Spain, ^5^BioScripts - Centro de Investigación y Desarrollo de Recursos Científicos, 41012 Sevilla, Andalusia, Spain, ^6^Institut Botànic de Barcelona (IBB-CSIC-ICUB), 08038 Barcelona, Catalonia, Spain and ^7^Laboratoire d’Evolution et Systématique, Université Paris Sud, UMR8079 CNRS-UPS-AgroParis-Tech, 91405 Orsay Cedex, France

## Abstract

Two plant genome size databases have been recently updated and/or extended: the Plant DNA C-values database (http://data.kew.org/cvalues), and GSAD, the Genome Size in Asteraceae database (http://www.asteraceaegenomesize.com). While the first provides information on nuclear DNA contents across land plants and some algal groups, the second is focused on one of the largest and most economically important angiosperm families, Asteraceae. Genome size data have numerous applications: they can be used in comparative studies on genome evolution, or as a tool to appraise the cost of whole-genome sequencing programs. The growing interest in genome size and increasing rate of data accumulation has necessitated the continued update of these databases. Currently, the Plant DNA C-values database (Release 6.0, Dec. 2012) contains data for 8510 species, while GSAD has 1219 species (Release 2.0, June 2013), representing increases of 17 and 51%, respectively, in the number of species with genome size data, compared with previous releases. Here we provide overviews of the most recent releases of each database, and outline new features of GSAD. The latter include (i) a tool to visually compare genome size data between species, (ii) the option to export data and (iii) a webpage containing information about flow cytometry protocols.

## INTRODUCTION

The total amount of DNA in the unreplicated haploid or gametic nucleus of an organism is referred to as the C-value or genome size (1), and across eukaryotes it varies approximately 66 000-fold (2). The smallest genome so far reported is found in the parasitic microsporidian *Encephalitozoon intestinalis* (3,4) with a C-value of just 2.3 Mb [C-values are usually reported either in terms of mass (picograms, pg, with 1 pg = 10^−12 ^g) or number of base pairs, with most estimates given in megabase pairs or gigabase pairs. N.B. 1 pg = 978 Mb (5).]. At the other end of the scale, the largest reliable genome size estimate is for the angiosperm *Paris japonica* with a C-value of 148 880 Mb (2). Interest in this genomic character goes back to the late 1940s and early 1950s when researchers started to systematically measure and compare DNA amounts within and between plants and animals (6–8). These early studies revealed that genome size was remarkably constant within a species (8), and provided support for DNA rather than protein being the hereditary material [reviewed in (9)]. Since then interest has remained high as genome size has been shown to be a key biodiversity character of fundamental biological and evolutionary significance (9–11). In addition, knowledge of genome size has practical implications, such as estimating the cost and time for whole genome sequencing projects (12), and selecting protocols for DNA fingerprinting studies (13,14).

Despite this realization of the importance of genome size to both fundamental and applied research, for many years it was difficult to know whether a genome size measurement existed for a particular taxon and if so where to find it. This was because values were either scattered in the literature or unpublished. Nevertheless, this impediment has now been largely overcome by the release of electronic databases for several major groups of eukaryotes (15,16): animals (http://www.genomesize.com), fungi http://www.zbi.ee/fungal-genomesize) and plants (http://data.kew.org/cvalues and http://www.asteraceaegenomesize.com). Together these databases currently contain data for >15 000 species comprising 4972 animals, 1581 fungi and 8922 plants.

Interest in the field of genome size research remains high and new genome size data continue to be published in the literature. Thus, keeping the databases up to date has necessitated the continued release of new versions. This article focuses on the two open-access plant genome size databases, which have recently been updated: the Plant DNA C-values database (Release 6.0, December 2012, http://data.kew.org/cvalues) and the Genome size in Asteraceae database (GSAD; Release 2.0, June 2013, http://www.asteraceaegenomesize.com).

## THE PLANT DNA C-VALUES DATABASE

**Figure 1.**
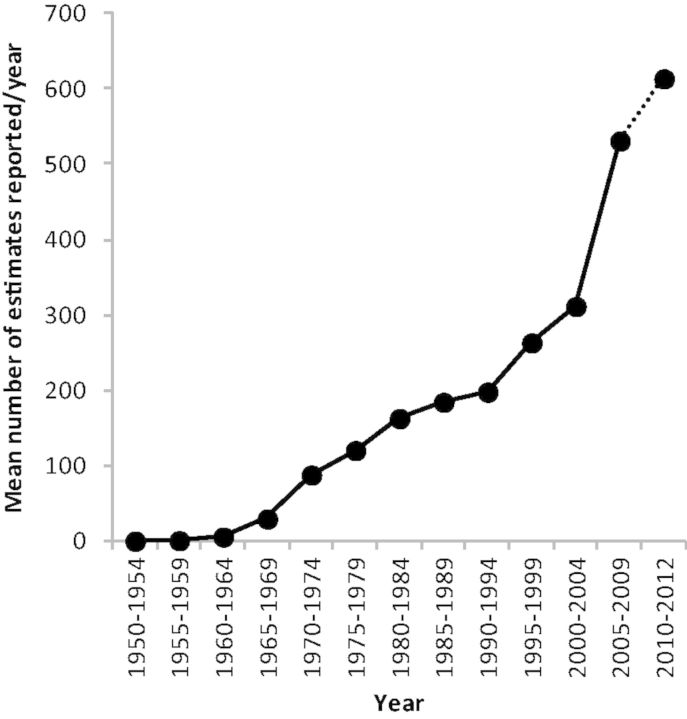
Mean number of plant genome size estimates reported per year over 12 successive 5-year periods and the 3-year period 2010–2012 (dotted line), between 1950 and 2012. Data taken from the Plant DNA C-values database (Release 6.0, December 2012).

**Figure 2.**
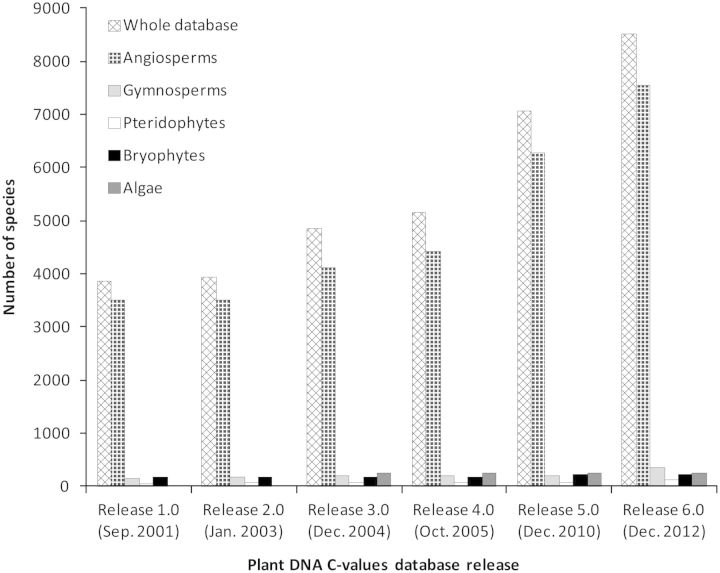
Growth of the Plant DNA C-values database in terms of the total number of species represented in the whole database (diagonal hatch) and for each individual group (squares = angiosperms; light gray = gymnosperms; white = pteridophytes; black = bryophytes; dark gray = algae).

The Plant DNA C-values database (http://data.kew.org/cvalues) was first launched in 2001 to provide a user-friendly searchable database where both published and unpublished values of plant genome size could be readily found (15,17). It contained data for 3864 species that had been compiled and published by Bennett and colleagues in hard copy between 1976 and 2000 (18–23). Since 2001, the increasing volume and rate of production of new data on plant genome sizes (Figure 1) has led to five further updates of the database, with the most recent release (Release 6.0, December 2012) containing data for 8510 species compiled from 808 original reference sources. The majority (89%) of estimates are for angiosperms (7542 species from 695 references), with the others comprising 365 gymnosperms (from 48 references), 128 pteridophytes (comprising monilophytes and lycophytes from 21 references), 232 bryophytes (from seven references) and 253 algae (from 37 references) (Figure 2). A detailed description of the organization, search options and output fields in the Plant DNA C-values database has already been given in (15) and is also available from the ‘Help’ web page of the database (http://data.kew.org/cvalues/searchguide.html). This outlines the diverse and flexible search options available to enable the user to interrogate the database. For example, the user can choose to (i) search the whole database, or just a subset of it (e.g. just angiosperms), (ii) restrict searches to a specific range of DNA amounts, chromosome numbers and/or ploidy levels, (iii) restrict searches to a particular family, higher order plant group and (iv) conduct wild card searches. In addition, the various options available for displaying the results of the search are given, such as the choice to output the data as 1C, 2C or 4C values in Mb or pg, and to sort the results by DNA amount, chromosome number, ploidy level or taxonomically (e.g. alphabetically by family, genus, species).

It is noted that the Plant DNA C-values database does not currently display information about which calibration standard has been used to estimate the genome size of a particular species, despite the realization that choice of standard and its assumed C-value are two of the major factors contributing to artifactual genome size variation, as outlined in Doležel and Greilhuber (24) and Suda and Leitch (25). Clearly there is a need to deal with these important issues and to reach a consensus on the selection of appropriate calibration standards and uniformity on the C-values assumed. However, as an interim measure, the option to display the standard species used will be included in the next release of the database.

### What is new in Release 6.0 of the Plant DNA C-values database

Compared with the previous release of the Plant DNA C-values database (i.e. Release 5.0, December 2010 with data for 7058 species), the number of species included has increased by 17%. Analysis shows that 2010–2012 had the highest rate of plant genome size data generation known (i.e. approximately 460 species not previously listed in the database (= ‘new’ species) added per year, and >600 estimates in total per year (Figure 1).

#### Angiosperms

Most of the novel additions to the database have come from research in angiosperms, where data for 1255 species not previously listed have been added. Not only has this increased the percentage of angiosperm species with genome size data to approximately 2.1% [based on an estimate of 352 000 angiosperm species in total, (26)], but representation at the generic and family levels has also improved. At the generic level, the new release includes estimates for 187 genera not previously listed and brings the number with at least one genome size estimate to 1635, corresponding to 12.6% of the 12 962 genera recognized (27). The database also includes genome sizes for 249 families, although only nine families not previously represented in the database were added in Release 6.0. Of the 415 families currently recognized (28), 60% have at least one genome size estimate.

#### Other land plant groups and algae

In other land plant groups, the most notable progress in improving genome size representation has been in the gymnosperms where the number of ‘new’ species has increased by 43%. This is largely due to several recent surveys by Zonneveld (29–31), which together have generated data for all cycad genera and 64 of the 69 conifer genera now recognized (32). Consequently, genome size data are now available for 35% of gymnosperm species (355 out of the 1026 species recognized by 32), including representatives of all 12 gymnosperm families, and 98% of the genera (81 out of 83 genera recognized by 32). Gymnosperms are the best represented of all land plant groups in terms of genome size (Table 1).

**Table 1.** Minimum (Min.), maximum (Max.) and mean 2C-values for each plant group represented in the Plant DNA C-values database (Release 6.0, Dec. 2012), together with percentage representation of species in each group

Plant group	Min. (pg)	Max. (pg)	Mean (pg)	Range (Max./Min.)	Approximate number of species recognized	Number of species in the Plant DNA C-values database	Approximate % species representation in the Plant DNA C-values database
Algae							
Chlorophyta	0.02	39.2	3.4	1960-fold	6500	91	1.4
Rhodophyta	0.02	2.8	0.8	140-fold	6000	118	1.9
Phaeophyta	0.2	1.8	0.8	9-fold	1500	44	2.9
Bryophytes							
Liverworts	0.42	15.94	2.4	38-fold	5000	48	0.9
Mosses	0.34	4.1	1	12-fold	12 000	184	1.5
Pteridophytes							
Lycophytes	0.18	23.94	4.8	133-fold	900	27	3
Monilophytes	1.54	145.36	25.6	94-fold	11 000	101	0.9
Gymnosperms	4.5	72	36.2	16-fold	1026	355	34.6
Angiosperms	0.13	304.46	11.6	2342-fold	352 000	7542	2.1

Progress in other land plant groups and algae remains poor, with the addition of only 46 pteridophyte species not previously included in the database and no new data for bryophyte or algal species. Nevertheless, this will be addressed in Release 7.0 planned for 2014 as new genome size data for the bryophyte groups liverworts [67 species from 33 families, (33)] and hornworts [24 species from 5 families, (34)] will be added, together with new data for algae [e.g. (35–38)] and other land plant groups collated from the literature.

### The Plant DNA C-values database provides insights into plant genome size diversity

**Figure 3.**
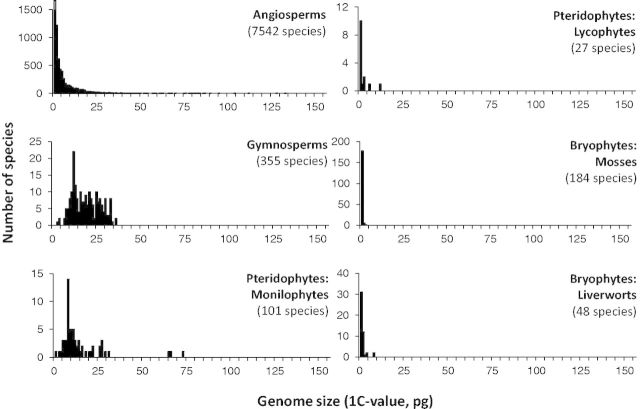
Histograms showing the distribution of genome sizes in the different plant groups using data taken from the Plant DNA C-values database (Release 6.0, December 2012).

Overall, analysis of the data available in the Plant DNA C-values database illustrates the considerable diversity in genome sizes between the different land plant and algal groups, both in terms of the range of genome sizes encountered and the distribution of genome sizes (Figure 3, Table 1). Such different genome size profiles highlight the contrasting genome size dynamics operating between plant lineages (39,40) and argue strongly for the need to continue to collate and analyze genome sizes across the plant tree of life to form a more holistic understanding of plant genomic diversity.

## THE GSAD

GSAD (http://www.asteraceaegenomesize.com) provides genome size data specifically for Asteraceae (Compositae), which are considered to be one of the largest plant families (24 000–30 000 species) with a worldwide distribution, except Antarctica. Overall, Asteraceae account for approximately 7–9% of angiosperm species on Earth and include many economically important representatives such as those used for food (e.g. artichoke—*Cynara cardunculus*, sunflower—*Helianthus annuus*), medicine (e.g. artemisinin, an active compound against malaria extracted from the sweet wormwood—*Artemisia annua*) and horticulture (e.g. *Chrysanthemum* and *Dahlia* species and hybrids), or which are invasive noxious weeds (e.g. *Taraxacum*). This family has been the target of numerous molecular systematic and genomic studies (e.g. 41–43) and the focus of evolutionary-developmental research such as floral development in *Gerbera* or *Helianthus* (44). The sunflower is also the subject of an ongoing whole genome sequencing project (45), with the current release containing data for >80% of the genome (45,46).

Development of GSAD was initiated by research groups based at the Universitat de Barcelona and Institut Botànic de Barcelona (IBB-CSIC-ICUB) in collaboration with a team from the Université de Paris Sud-CNRS (http://www.etnobiofic.cat). It arose from their long-term scientific interest in Asteraceae, particularly from a genome size perspective (16,47–51). Given the large amounts of genome size data for Asteraceae generated by these and other research groups, the decision to develop and curate an online genome size database focused specifically on Asteraceae was taken. The aim was to complement the Plant DNA C-values database in the same way that the Index to Chromosome Numbers in Asteraceae (http://www.lib.kobe-u.ac.jp/infolib/meta_pub/G0000003asteraceae_e) complements the more general Index to Plant Chromosome Numbers (http://www.tropicos.org/Project/IPCN). Additionally, GSAD provides data for hybrid taxa, varieties, forms and cultivars of Asteraceae, which are not usually included in the Plant DNA C-values database [e.g. see (10,17)].

**Figure 4.**
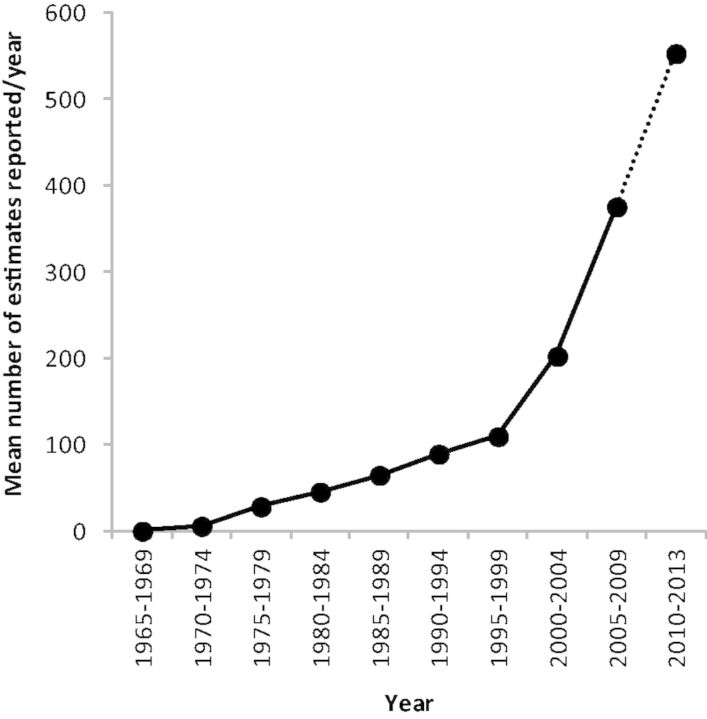
Mean number of Asteraceae genome size estimates reported per year over 9 successive 5-year periods and the 4-year period 2010–2013 (dotted line), between 1965 and 2013. Data taken from GSAD (Release 2.0, June 2013).

GSAD was launched in July 2010 (Release 1.0) and a detailed description of its content and organization is given in Garnatje *et al.* (16). In the 3 years since the first release, data for a further 412 species have been collated from 40 publications (either already published or in press by June 2013) reflecting both the continued scientific interest in the field and the inclusion of previously overlooked articles (Figure 4). These new data have been incorporated into the new release (Release 2.0, June 2013), which contains genome sizes for 1219 species [Currently GSAD contains C-value data for approximately 400 species of Asteraceae not listed in the Plant DNA C-values database (Release 6.0, December 2012). Many of the additional species are from unpublished data and hence were not available for inclusion in the Plant DNA C-values database. In addition, GSAD includes some data that have been published or were in press in 2013 and hence were not included in the 2012 release of the Plant DNA C-values database.], 186 genera, 20 tribes and six subfamilies compiled from 133 original references. Currently, GSAD is the only genome size database focused on a single plant family.

### Database content update

Overall, the total number of species and genera listed in GSAD has grown by 51 and 72%, respectively. In addition, Release 2.0 now includes some well-known genera such as *Leontopodium* and *Mutisia* for which no previous records were available. Table 2 provides information on the percentage of species with genome size data for the 6 subfamilies and 20 tribes comprising Asteraceae, together with their minimum, maximum, mean and range of C-values. With respect to Release 1.0, the most studied genera from a genome size perspective are still the same (Table 3), although *Hieracium* has moved from third to second position. Given the increasing rate at which new genome size data are being generated (Figure 4), it is clear that interest in this key biodiversity character in Asteraceae remains high and indeed, seems likely to increase in the coming years.

**Table 2.** Minimum (Min.), maximum (Max.) and mean 2C-values for each of the subfamilies (in bold) and tribes of Asteraceae represented in GSAD (Release 2.0, June 2013) together with percentage representation of species in each group

Subfamily and tribe	Min. (pg)	Max. (pg)	Mean (pg)	Range (Max./Min.)	Approximate number of species recognized^a^	Number of species in GSAD	Approximate % representation of species in GSAD
**Asteroideae**	**0.79**	**142**	**8.54**	**180-fold**	**15 500**	**676**	**4.4**
Anthemideae	2.56	142	11.16	55-fold	1800	308	17.1
Astereae	0.91	21.43	3.84	24-fold	3080	63	2
Calenduleae	1.75	5.41	3.16	3-fold	270	7	2.6
Coreopsidae	1.5	56.56	8.04	38-fold	550	31	5.6
Eupatorieae	2.2	7.2	3.64	3-fold	2200	8	0.4
Gnaphalieae	1.11	15.5	2.84	14-fold	1240	27	2.2
Heliantheae	2.14	30.54	8.78	14-fold	1500	46	3.1
Inuleae	1.12	7.34	2.3	7-fold	687	40	5.8
Madieae	2.8	3.13	2.96	1-fold	>200	2	1
Millerieae	0.98	11.5	5.24	12-fold	400	29	7.25
Polymnieae	5.4	5.4	5.4		3	1	33.3
Senecioneae	0.79	52.3	7.5	66-fold	3500	113	3.2
Tageteae	2.4	2.4	2.4		270	1	0.4
**Barnadesioideae**					**91**	**1**	**1.1**
Barnadesieae	8.44	8.44	8.44		91	1	1.1
**Carduoideae**					**>2600**	**229**	**8.8**
Cardueae	1.28	28.94	3.64	22-fold	2360	229	8.8
**Cichorioideae**	**0.8**	**39.9**	**6.56**	**50-fold**	**>2900**	**305**	**10.5**
Cichorieae	0.8	31.3	6.5	39-fold	>1500	282	18.8
Vernonieae	2.04	39.9	8.44	20-fold	>1000	23	2.3
**Gochnatioideae**					**88**	**1**	**1.1**
Gochnatieae	2.27	4.53	3.4		88	1	1.1
**Mutisioideae**	**3.66**	**7.8**	**6.26**	**2-fold**	**630**	**9**	**1.4**
Mutisieae	5.12	7.68	6.46	2-fold	200	6	3
Nassauvieae	3.66	7.8	5.9	2-fold	300	3	1

^a^Number of species recognized in each subfamily and tribe taken from Kubitzki (52) and Funk *et al.* (53).

**Table 3.** A comparison of the number of records for the most widely represented genera in the GSAD database

	Release 1.0	Release 2.0	Increase (%)
*Artemisia*	246 (1)	366 (1)	48.8
*Hieracium*	175 (3)	260 (2)	48.6
*Senecio*	185 (2)	198 (3)	7.0
*Helianthus*	97 (4)	157 (4)	61.9
*Crepis*	113 (5)	135 (5)	19.5

The ranking of the best represented genera is in brackets.

### Web interface features new to Release 2.0

Release 2.0 of GSAD includes several new features to enhance the user’s experience.

- A genome size representation tool is now included to enable the user to visually compare genome sizes for a set of species. This allows genome size differences within a given search output to be easily compared. A bar, whose size is directly proportional to genome size, is shown next to the genome size value of the species, together with a red line representing the mean value of the genus.
- Following the recommendations of Bateman on how to improve the usability of a database (54), another novel feature is the option to export data from a search to an Excel™ file, and/or email the results.

### New page tabs

Several new page tabs have been created for the new version. (i) ‘Publications’ provides a complete list of source references, together with a link to the pdf of the article (accessible if the user/user’s institution has permission, otherwise only the abstract is shown) if available, as some publications only exist in hard copy. (ii) ‘Protocols and Reagents’ contains information on how to estimate plant genome size by flow cytometry and links to books on these topics. The aim of this new tab is to help and guide scientists on how to use flow cytometry to estimate plant genome size accurately. (iii) ‘Help’ has notes on simple and advanced search options, a table on the methods used to estimate genome size and an explanation on how the genome size representation tool works. (iv) ‘News’ is a blog with information on upcoming meetings, relevant articles and links related to genome size and Asteraceae. (v) ‘Submit your Data’ provides the option for researchers to send data through a submission form. (vi) ‘What’s new?’ gives details of updates and improvements in each new release of the database.

### Updates to existing page tabs

Some tabs in Release 1.0 have been updated. For example, the ‘Home’ tab has a shorter introduction but now includes graphs to illustrate data increments from the first to the second release in terms of total number of estimates, species, genera and references. In addition, the number of estimates determined by different measurement techniques (e.g. flow cytometry, Feulgen microdensitometry) is given.

On the ‘How to cite?’ page, there is now a link to the pdf of Garnatje *et al.* (16) outlining the first release of GSAD (accessible if the user/user’s institution has permission). Finally, the ‘Links’ tab has been expanded to include links to other sites containing genome size data and related genomic information.

### Future prospects

The second release of GSAD arose from a considerable compilation effort and has led to a significant increase in the number of Asteraceae species with genome size data. Given this remarkable growth of data in recent years, annual updates are planned so that readily accessible global knowledge on Asteraceae genome sizes remains up to date. Other improvements to GSAD in the near future are likely to include the incorporation of links to published molecular phylogenetic and sequence data for species listed in any given search output, together with data for closely related genera, if available.

Despite the many species already listed, there are still conspicuous and important gaps in the knowledge of genome size in this large family. Species representation only stands at approximately 5%, and C-values are missing for most tribes (approximately 60%) and for 6 of the 12 recognized subfamilies. Nevertheless, the construction of this database has enabled such gaps to be highlighted and will hopefully encourage the development of working strategies to fill them. In this regard, the following 5-year targets are proposed to improve representation of genome sizes in Asteraceae: to estimate a further 1200 species, 130 genera, 10 tribes and 6 subfamilies to raise taxonomic representation to approximately 10% of species, approximately 20% of genera, approximately 70% of tribes and 100% of subfamilies by 2018.

## FUNDING

Royal Botanic Gardens, Kew, the Research Council of Norway [196468/V40]; the Spanish government [CGL2010-22234-C02-01, -02/BOS]; Catalan government [2009SGR00439]; ‘Juan de la Cierva’ postdoctoral contracts [JCI-2011-10124 to S.G. and JCI-2010-9432 to O.H.]; A ‘Beatriu de Pinós’ postdoctoral contract [BP-2011-A-00292 to J.P.]; Universitat de Barcelona [ADR-2011-38 to G.M.]; Ministerio de Educación of the Spanish government [AP2008-03441 to D.V.]. Funding for open access charge: The Royal Botanic Gardens, Kew; the Universitat de Barcelona [AO 161]; the Spanish government [CGL2010-22234-C02-01, -02/BOS] and the Catalan government [2009SGR00439].

*Conflict of interest statement*. None declared.
